# XYomics: detecting sex-dependent molecular mechanisms in omics data

**DOI:** 10.1093/nar/gkag759

**Published:** 2026-07-30

**Authors:** Sophie Le Bars, Mohamed Soudy, Enrico Glaab

**Affiliations:** Biomedical Data Science Group, Luxembourg Centre for Systems Biomedicine (LCSB), University of Luxembourg, 6 Avenue du Swing, Belvaux 4367, Luxembourg; Biomedical Data Science Group, Luxembourg Centre for Systems Biomedicine (LCSB), University of Luxembourg, 6 Avenue du Swing, Belvaux 4367, Luxembourg; Biomedical Data Science Group, Luxembourg Centre for Systems Biomedicine (LCSB), University of Luxembourg, 6 Avenue du Swing, Belvaux 4367, Luxembourg

## Abstract

Understanding sex-dependent differences in disease risk, manifestation, and treatment response is essential for precision medicine. While funding agencies now mandate consideration of Sex as a Biological Variable (SABV), existing bioinformatics tools lack systematic approaches to characterize sex-related molecular mechanisms. Current practices frequently treat sex as a confounding variable, which may obscure important biological differences such as sex-specific alterations, sex-dimorphic changes (opposite effects between sexes), and sex-modulated changes (different effect magnitudes). We present XYomics, an open-source R package for systematic analysis of sex-dependent alterations in biomedical omics data. The software identifies sex-specific, sex-dimorphic, and sex-modulated changes at both individual feature and systems levels. XYomics implements dual analytical modes: sex-disease interaction term modeling for adequately powered datasets and sex-stratified analysis with robust non-significance filtering for smaller sample sizes. Using single-cell RNA sequencing data from Alzheimer’s disease patients, we demonstrate how XYomics identifies sex-dimorphic genes largely undetected by standard sex-averaged analyses. By integrating statistical categorization with pathway enrichment and network analysis using a curated hormone signaling interactome, the software facilitates discovery of sex-specific biomarkers and disease mechanisms frequently obscured in sex-aggregated analyses.

## Graphical Abstract

**Graphical Abstract:**
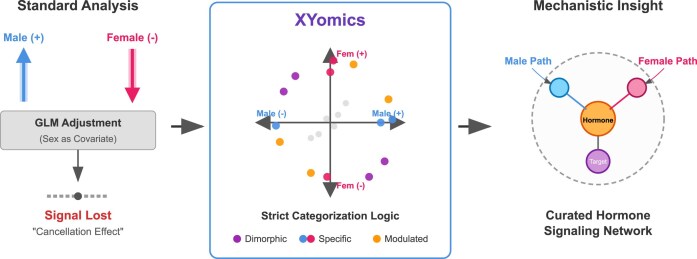

## Introduction

### The relevance of Sex as a Biological Variable (SABV)

One of the most common challenges in preclinical biomedical research is imbalanced consideration of the sexes. In the past, a majority of animal studies and early-phase clinical trials have been conducted exclusively on male subjects, in order to avoid the perceived variability introduced by the estrous cycle (in animals) and the menstrual cycle (in humans). This practice has led to significant gaps in our understanding of female biology and has likely contributed to higher rates of adverse drug reactions in women [1]. In response, major funding bodies, including the US National Institutes of Health (NIH) and the European Commission, have instituted mandates requiring the consideration of Sex as a Biological Variable (SABV) in all funded research [2]. Nonetheless, adherence to these SABV mandates remains limited. Sex-based analyses are still infrequently performed in preclinical and basic science studies, and when they are conducted, they are often flawed or misreported [3, 4]. For instance, Garcia-Sifuentes and Maney [5] found that the majority of NIH-funded studies claiming to examine sex as a biological variable did not employ appropriate statistical tests for sex differences, either by simply including both sexes without formal comparisons or by using inadequate methods.

However, complying with these mandates requires more than simply including both sexes in a study. It also necessitates analytical tools that can detect and interpret the complex ways in which sex interacts with disease biology. Sex differences are not merely binary; they arise from a complex interplay of chromosomal effects (e.g. X-chromosome inactivation escape), organizational and activational effects of sex hormones (estrogens, androgens, and progestogens), and immune system dimorphism [6–8]. In diseases such as Alzheimer’s disease (AD), autoimmune disorders (e.g. Lupus and Multiple Sclerosis), and cardiovascular disease, these differences manifest not just as differences in risk, but as distinct molecular etiologies [9, 10].

### The cancellation effect in standard bioinformatics

Despite the biological imperative to consider sex-dependent mechanisms, the standard bioinformatics toolkit has not evolved to explicitly model these complexities. In a typical RNA-seq or proteomics workflow using standard packages such as limma [11] or DESeq2 [12], sex is usually handled in one of two ways, both of which are suboptimal for discovery:

1. **Sex-adjustment:** Researchers fit a generalized linear model (GLM) of the form $Expression \sim Condition + Sex$, assuming disease effects are identical in both sexes. However, if a gene is overexpressed in males (+2 logFC) but underexpressed in females (−2 logFC), the average effect is zero and the gene is reported as non-significant.
2. **Simple overlap:** Researchers analyze sexes separately and overlay the resulting significant gene lists. This approach suffers from the “threshold effect.” If a gene has a *p*-value of 0.049 in males and 0.051 in females, it is classified as “male-specific,” even though there is no statistical difference between the two groups. This leads to a high rate of false-positive sex-specific claims that fail replication.

### The XYomics approach

To address these methodological limitations, we developed XYomics, a comprehensive R framework designed to standardize and facilitate the discovery of sex-dependent mechanisms in functional omics data. XYomics shifts the analytical focus from “controlling for sex” to “interrogating sex.” Key aspects of the software include:

#### Strict categorization logic and interaction modeling

**Figure 1.**
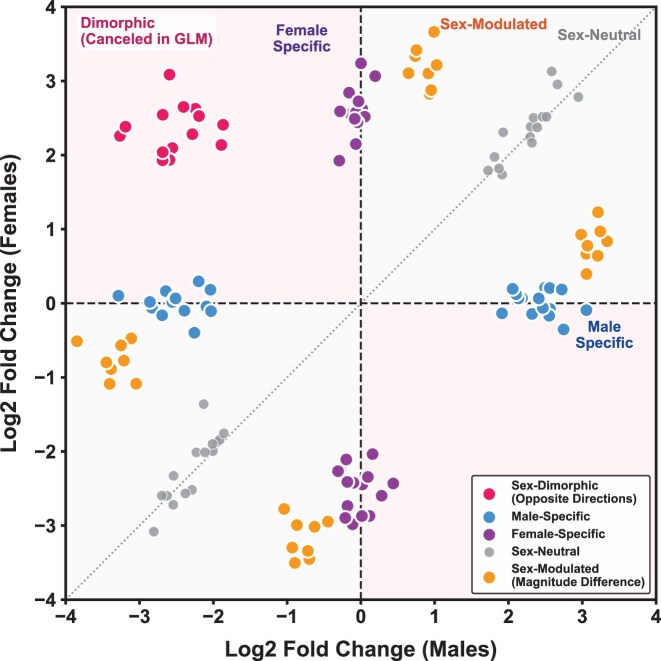
Conceptual framework of sex-dependent categorization. A comparison of log2 fold changes in males versus females illustrates the distinct categories identified by XYomics. Conventional GLMs typically average the values within the “sex-dimorphic” quadrant (red), yielding an overall fold change close to zero, and do not capture magnitude differences in the “sex-modulated” quadrant. Sex-modulated genes are detected exclusively through interaction analysis (Mode 1), whereas sex-specific and sex-dimorphic genes can be identified by either the stratified (Mode 2) or interaction modeling approach. XYomics is specifically designed to recover such signals.

A formal decision tree uses both significance and non-significance criteria to robustly classify genes as sex-specific or sex-dimorphic (opposing directions, see Fig. 1). Additionally, XYomics explicitly identifies sex-modulated genes (magnitude differences) through formal sex-disease interaction modeling.

#### Mechanistic context

Unlike tools that stop at gene lists, XYomics includes a proprietary, manually curated network of hormone signaling interactions (nuclear receptors, steroidogenic enzymes, and their cofactors). This allows users to immediately map transcriptomic changes to potential upstream hormonal drivers for mechanistic data interpretation.

#### Multi-omics support

The package is built on S3 classes compatible with both bulk (matrices) and single-cell (Seurat) data structures, allowing it to serve as a unified approach for diverse data types.

## Materials and methods

### Software architecture and design philosophy

The main design motivation for XYomics was to eliminate fragmented workflows that currently characterize sex-aware analyses, where researchers must manually coordinate between differential expression tools, custom filtering scripts, and separate pathway/network analysis packages. By unifying these steps into a single analytical object that carries forward all intermediate results, XYomics ensures that downstream interpretations (e.g. pathway enrichments and network analyses) remain automatically synchronized with the upstream statistical categorizations, reducing both user error and the risk of analytical inconsistencies.

**Figure 2.**
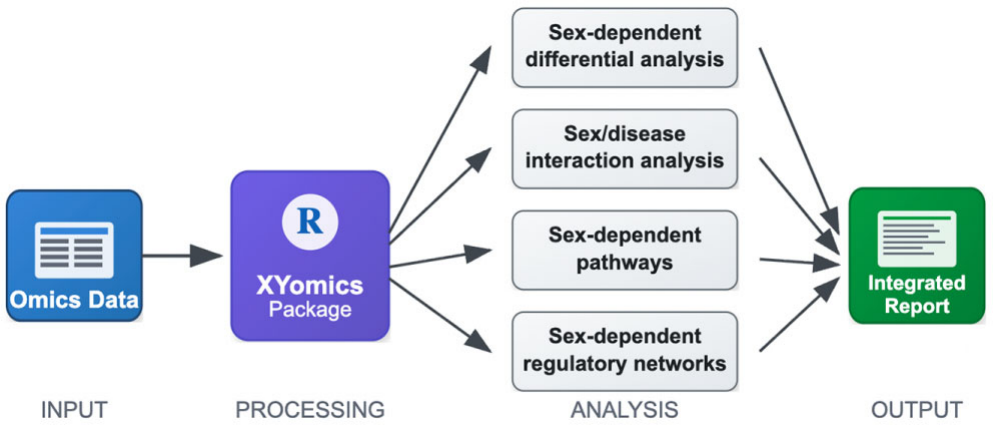
Workflow overview. The pipeline takes preprocessed omics data as input and includes sex-dependent differential expression analysis, sex-disease interaction analysis, sex-dependent pathway analysis, and gene regulatory network analysis. All analysis outputs are combined into an integrated report with visualizations and ranking tables.

The workflow is structured into four sequential modules, which can be run end-to-end or independently (see Fig. 2).

#### Data preprocessing module

The entry point of the pipeline is designed to be flexible. XYomics accepts transcriptomics data as normalized log-expression matrices and raw counts (via DESeq2/edgeR), single-cell objects through direct input of Seurat objects (allowing users to perform sex-dependent analysis on specific cell clusters such as astrocytes or microglia without complex data extraction steps), and proteomics/metabolomics data as both normalized/log-transformed intensity matrices with associated metadata.

Upon data input, the software performs automated internal quality control (which can also be run independently via the validate_input_sc() or validate_input_bulk() functions), ensuring that sex labels are consistent, that sufficient replicates exist for stratified analysis ($N \ge 3$ per group recommended), and that data are appropriately normalized. For single-cell data, cells from the same individual are not independent biological replicates, so statistical power should be assessed at the level of donors rather than cells. Accordingly, when an optional donor/sample column is provided via the donor_col argument, validate_input_sc() additionally counts the number of unique donors per sex $\times$ phenotype group and warns when this falls below the recommended minimum; when no donor column is supplied, the function reports cell-level counts and explicitly warns that biological replication could not be assessed. Furthermore, the software allows the user to account for sparsity by selecting specialized statistical tests (e.g. MAST [13]) that are suitable for zero-inflated data.

#### The statistical categorization approach

This core module of XYomics performs the differential analysis using two alternative strategies designed to accommodate different dataset sizes:

**Strategy A: Interaction modeling (large *N*)**. For datasets with sufficient statistical power, the software fits a linear model including a sex-disease interaction term. This approach explicitly captures the difference in disease response between sexes as a model coefficient.

**Strategy B: Stratified analysis with robust filtering (small *N*)**. Recognizing that many biomedical omics datasets are derived from small cohorts, with insufficient sample size for robust interaction modeling, XYomics implements a stratified analysis with a dual-threshold filter. Unlike standard overlap analyses that suffer from high false-positive rates due to threshold effects, this strategy requires that a gene be statistically significant in one sex and robustly non-significant in the other to be classified as sex-specific (i.e. using two dedicated threshold *P*-values for significance and non-significance). The thresholds are fully customizable by the user, though the package employs conservative default values to ensure robustness.

The mathematical definitions for these categories are detailed below in the Statistical Methodology section.

#### Systems biology integration module

Once genes are categorized, XYomics maps them to pathway and network structures that encode prior biological knowledge for systems-level analysis.

**Pathway analysis:** This module tests for sex-dependent gene/protein set over-representation in the databases Gene Ontology (GO, [14]), KEGG [15], Reactome [16], and other user-provided pathway resources. Importantly, implementing enrichment analyses in a sex-aware manner ensures that all categories of sex differences (sex-specific, sex-dimorphic, or sex-modulated) are considered to answer pattern-specific interpretative questions (e.g. “Which pathways show sex-dimorphic changes?”).

**Hormone network integration:** The package contains an embedded network graph object representing a comprehensive human hormone signaling network. This network was assembled by combining genes from hormone-related pathways in the GO and KEGG databases and constructing directed interaction networks using the direct interaction algorithm in the software GeneGo MetaCore^™^. In combination with network analysis functions and topological metrics implemented in the package, this allows XYomics to identify coordinated, sex-dependent network alterations and detect which hormonal hubs are most central to the observed sex-dependent changes.

**Global protein network:** The package also provides easy programmatic access to global protein–protein interaction (PPI) networks using molecular interaction data from the database STRING [17], supporting genome-scale network analyses of sex-dependent changes in omics data.

#### Automated reporting module

To ensure reproducibility, XYomics includes a reporting module using R Markdown. The function generate_cat_report() takes the results from an analysis and renders a standalone HTML file. This report includes volcano plots color-coded by significance and separated by sex-dependent category, interactive tables (sortable and searchable lists of sex-specific targets), and network graphs.

### Statistical methodology

As outlined above, XYomics offers two complementary analytical strategies, *Interaction Analysis* (optimized for sufficiently large samples, with at least $N \geqslant 20-30$ per group and a practical minimum of 50 in total, where *N* denotes the number of biological replicates, i.e. donors in single-cell data rather than cells) and *Stratified Analysis* (optimized for small sample sizes). Here, we provide the formal statistical definitions underlying each mode.

#### Mode 1: Interaction term modeling

For datasets with sufficient statistical power, XYomics fits a unified linear model including an interaction term. Let $Y_{gi}$ be the expression of gene *g* in sample *i*:


(1) \begin{eqnarray*}
Y_{gi} &=& \beta _0 + \beta _1 X_{dis,i} + \beta _2 X_{sex,i} + \beta _3 (X_{dis,i} \times X_{sex,i}) + \epsilon _i\\
\end{eqnarray*}

Where $X_{dis}$ is the disease status (0 = Control, 1 = Disease) and $X_{sex}$ is the sex (0 = Female, 1 = Male).

In this framework, $\beta _1$ estimates the disease effect in females (the reference sex), and $\beta _3$ estimates the difference in disease effect between males and females, i.e. the male-specific disease effect minus the female-specific disease effect. Equivalently, the software estimates this interaction through the difference-in-differences contrast $(\mathrm{Disease}_{\mathrm{Male}} - \mathrm{Control}_{\mathrm{Male}}) - (\mathrm{Disease}_{\mathrm{Female}} - \mathrm{Control}_{\mathrm{Female}})$, so that a positive interaction coefficient indicates a stronger disease effect in males and a negative coefficient a stronger disease effect in females. A gene is classified as sex-modulated if the interaction coefficient $\beta _3$ is statistically significant (FDR $< 0.05$). This is the method of choice for detecting magnitude differences (e.g. logFC of 2.0 in males versus 4.0 in females).

#### Mode 2: Stratified analysis with robust filtering

For datasets with small numbers of samples and insufficient statistical power to robustly estimate sex-disease interactions, this approach fits separate statistical models for males (*M*) and females (*F*). For bulk data (e.g. microarray, proteomics, and metabolomics), we integrate limma’s empirical Bayes moderated *t*-statistic within a sex-stratified inference framework that jointly evaluates significance and non-significance criteria across both sexes to enable robust categorization. For single-cell RNA-seq data, the user can select the differential expression method most appropriate for their dataset via the test.use parameter, choosing among MAST, bimod, poisson, LR, negbinom, or DESeq2 (i.e. any test in Seurat::FindMarkers() that returns *P*-values and a log-fold change). By default, the package implements a robust rank-based test (Wilcoxon Rank Sum). Alternatively, zero-inflated models, such as MAST, can be used within the same analytical framework; the MAST test is provided through the dedicated Bioconductor package MAST, invoked via Seurat::FindMarkers(), and is listed under the package Suggests, so it is installed on demand for users who select test.use = ‘MAST’.

To categorize a gene *g* as sex-specific, we apply a dual-threshold logic involving a significance threshold ($\alpha$, default: adjusted *P*-value ($p_{adj}$) $<$ 0.05) for the target sex and a non-significance threshold ($\beta$, default: nominal *P*-value $>$ 0.5) for the other sex. Note that $\alpha$ typically incorporates not only a significance threshold but also a minimum log-fold change requirement (default 0.25), ensuring that selected genes carry biological magnitude. The multiple testing correction is applied independently within each sex-specific differential expression model prior to dual-threshold categorization.

**Definition of female-specificity (analogous for male-specificity):**


(2) \begin{eqnarray*}
\mathrm{FemaleSpec}_g = (p_{adj, F} < \alpha ) \wedge (p_{nom, M} > \beta )
\end{eqnarray*}

This condition ensures that the gene/protein expression is significantly altered in females, while showing no statistical evidence of change in males (*P*-value is large).

**Definition of sex-dimorphism:**


(3) \begin{eqnarray*}
\mathrm{Dimorphic}_g &= & (p_{adj, M} < \alpha ) \wedge (p_{adj, F} < \alpha ) \\&& \wedge (\mathrm{sign}(\mathrm{logFC}_M) \ne \mathrm{sign}(\mathrm{logFC}_F))
\end{eqnarray*}

This condition ensures that the gene/protein expression is significantly altered in both sexes, but with opposite directions of the change.

### Hormone signaling network construction

To provide biological context for the analysis of sex differences in omics data, we created a specialized knowledge graph, the **XYomics Hormone Signaling Network**. This was assembled via a multi-step curation process:

In the first **seed selection step**, we queried the Gene Ontology (GO) and KEGG databases for all terms and pathways related to sex hormone activity (e.g. “cellular response to estrogen stimulus,” “androgen receptor signaling pathway,” and “progesterone-mediated oocyte maturation”). For reference, Supplementary Table S1 lists the parent hormone-related GO terms and KEGG pathways used to construct the network; all child GO terms associated with these parent GO terms were also included in the construction. A summary of the contents of the resulting network is provided in Supplementary Table S2.

In the second **interaction expansion step**, using the commercial GeneGo MetaCore™ database, we retrieved experimentally confirmed, directed PPIs. Specifically, we applied the direct interaction algorithm to identify the immediate network neighbors of the seed genes. Only interactions annotated with the following mechanisms were retained: binding, transcription regulation, influence on expression, co-regulation of transcription, and regulation. All interactions were retained as undirected for the analyses presented here, although directionality is preserved in the network object. This captures the core nuclear receptors (e.g. *ESR1, AR*) and their immediate coactivators/corepressors (e.g. *NCOA1, NCOR1*).

In the third **topology analysis step**, the resulting network was imported into R using the igraph package. We pre-calculated centrality metrics (betweenness, degree) to identify structural hubs.

### Active subnetwork identification

To identify the subnetwork with the strongest alterations in a given experiment, we apply the Prize-Collecting Steiner Forest (PCSF) algorithm [18]. The problem is modeled as searching a “forest” subgraph $F(V_F, E_F)$ within the global hormone network $G(V, E)$ that minimizes the objective function:


(4) \begin{eqnarray*}
\mathrm{Obj}(F) = \sum _{e \in E_F} c(e) + \lambda \sum _{v \notin V_F} p(v) + \omega \cdot \kappa
\end{eqnarray*}

Where $p(v)$ is the “prize” for including a node (derived from the gene’s sex-specific significance score or absolute logFC between the disease and control condition), $c(e)$ is the “cost” of including an edge (derived from interaction confidence as defined in the STRING database, $1 - (\mathrm{conf}/max(\mathrm{conf}))$), $\kappa$ is the number of trees in the forest, and $\lambda$ and $\omega$ are tuning parameters balancing the trade-off between network size and node inclusion.

This optimization identifies coordinated subnetwork alterations and associated high-confidence “linker” genes (also called Steiner nodes) that may not be differentially expressed themselves but are structurally required to connect the observed sex-dependent differential genes/proteins.

### Case study: Identifying sex-dimorphic changes in Alzheimer’s disease

To illustrate the analytical capabilities of XYomics, we performed a comprehensive re-analysis of a single-nucleus RNA sequencing (snRNA-seq) dataset from the entorhinal cortex of AD patients and age-matched healthy controls [19] (GEO accession: GSE138852). This dataset was selected because neurodegenerative diseases are known to show a strong sex-dependent prevalence and pathology, while the specific molecular drivers remain elusive.

The dataset includes transcriptional profiles from 12 individuals (AD: 4 males, 2 females; Control: 4 males, 2 females). We analyzed multiple brain cell populations, including astrocytes (*N* = 2595 cells), microglia, neurons, oligodendrocytes, and oligodendrocyte precursor cells, to capture cell type–specific patterns of sex-dimorphic gene expression. These cell types play complementary roles in neuroinflammation, metabolic support, and myelination processes that are disrupted in neurodegenerative disorders and are known to be influenced by sex-dependent factors, including hormonal signaling [20, 21]. Detailed results for each cell type are provided in Supplementary Tables S3–S13 and Supplementary Figs S1–S11.

#### Preprocessing and quality control

Raw sequencing data were processed using a standard Seurat v5 workflow. Specifically, quality control filtering included removing nuclei with $< 200$ detected genes or $> 20\%$ mitochondrial reads (indicative of cellular stress or membrane rupture). Gene expression counts were normalized using the LogNormalize method with a scale factor of 10 000. To mitigate donor-specific batch effects, which can often confound sex-specific signals in small cohorts, we integrated the data using the standard Seurat integration workflow [22]. The resulting batch-corrected embedding was used for clustering, and the cell type clusters were isolated using the expression of canonical marker genes (see Supplementary Materials).

### Software and algorithm availability

The XYomics package is implemented in the statistical programming language R (compatible version $\ge$ 3.6, recommended $\ge$ 4.3.2) and is freely available without registration at https://gitlab.com/uniluxembourg/lcsb/bds/xyomics under the MIT license. This GitLab repository also includes the source code, documentation, and vignettes for bulk and single-cell RNA-seq workflows. In addition, it provides example applications on two real datasets (proteomics and single-cell RNA-seq), which are included in the repository. The package imports the Bioconductor packages DESeq2, limma, edgeR, clusterProfiler, ReactomePA, AnnotationDbi, org.Hs.eg.db, and S4Vectors, and the CRAN packages Seurat, SeuratObject, igraph, ggplot2, ggraph, ggrepel, dplyr, tidyr, data.table, patchwork, cowplot, Rcpp, grid, and methods. Optional functionality (interactive tables and HTML reporting) additionally uses DT, kableExtra, knitr, rmarkdown, htmltools, gridExtra, R.utils, and stringr (declared under Suggests). Installation tools such as devtools or remotes are described in the package README. Minimum hardware requirements are 8 GB RAM and 2 CPU cores. The software was tested on Ubuntu 20.04, macOS Tahoe (Version 26), and Windows 11.

## Results

### The “cancellation effect”: a quantitative comparison

The primary motivation behind our work is that standard statistical models may obscure sex-dimorphic effects. To quantify this, we performed a head-to-head comparison between XYomics and a standard Generalized Linear Model (GLM) approach.

#### Standard approach

We fitted a GLM using the design formula $\sim \mathrm{Disease} + \mathrm{Sex}$. This model estimates the effect of Disease while holding Sex constant (effectively averaging the male and female responses).

#### XYomics approach

We used the sex_stratified_pipeline_sc() function which directly runs on all cell types in the XYomics package with the Stratified Analysis mode and the standard dual-threshold filter ($\alpha _{adj} = 0.05, \beta _{nom} = 0.5$).

The results revealed a striking loss of information in the standard approach. XYomics identified 306 sex-dimorphic genes (the complete list of sex-dependent differentially expressed genes (DEGs) in oligodendrocytes is provided in the GitLab repository, and the top 25 results are listed in Supplementary Table S6, with corresponding volcano plots shown in Supplementary Fig. S2). These are genes where the direction of the expression level change between AD patients and controls is opposite in males versus females (e.g. increased expression in males and decreased expression in females). When these 306 genes were assessed in the standard GLM, 228 (75%) failed to reach significance (FDR $> 0.05$).

These findings mathematically demonstrate the common cancellation effect in standard statistical analyses: when a gene is overexpressed in one sex (e.g. +1.5 logFC) and underexpressed with a similar absolute effect size in the other (e.g. −1.5 logFC), the aggregate effect size approaches zero. Consequently, standard pipelines discard these genes as “noise,” whereas XYomics correctly identifies them as high-priority candidates for sex-dimorphic changes.

### Biological interpretation: microglia display a female-specific inflammatory response

For illustration purposes, we applied the entire analysis pipeline to microglial cells. This revealed a strong female-specific transcriptional signature, with several immune-related pathways significantly enriched among female DEGs. For example, the “Efferocytosis” pathway (hsa04148, adj. $p = 1.18 \times 10^{-2}$) was among the top enriched categories. This enrichment reflects differential expression of genes associated with apoptotic cell clearance and phagocytic processes, including complement components (*C1QA, C1QB*), adhesion and immune interaction genes (*ITGB2, CDH1*), and immune-related modulators (*PTPN6, CSF2*).

These results indicate sex-associated differences in microglial gene expression related to pathways involved in cellular clearance and immune function. Although functional implications require further validation, these findings are consistent with previously reported sex differences in microglial transcriptional states in the context of AD [23]. Results for other cell types are provided in the Supplementary Materials.

### Mechanistic hypothesis generation: hormonal network links sex-dimorphism in oligodendrocytes to longevity pathways

While pathway analysis identifies broad biological processes, it does not explain the upstream regulatory mechanisms driving these changes. To illustrate XYomics’ capabilities for mechanistic hypothesis generation, we further studied the 306 sex-dimorphic genes identified in oligodendrocytes and applied the XYomics network analysis module, which builds on the Prize-Collecting Steiner Forest (PCSF) algorithm [18] within our curated hormone signaling network. Pathway enrichment analysis of oligodendrocyte-specific dimorphic genes identified the “Longevity regulating pathway” (hsa04213) (adjusted $p = 3.32 \times 10^{-2}$), suggesting an association between sex-dependent transcriptional regulation and cellular aging processes. Next, using these dimorphic genes as terminal nodes within a hormone-related interaction network, the PCSF algorithm identified a minimal subnetwork comprising the genes *PIK3R1, AKT3, HSPA1A*, and *HSP90AA1*. Notably, both *PIK3R1* and *AKT3* were over-expressed in females and under-expressed in males, indicating a sex-dependent shift in pathway activity. As central components of the PI3K–AKT signaling axis and the longevity pathway, these genes define a compact AKT-centered sub-pathway that links signal transduction with proteostasis.

**Figure 3.**
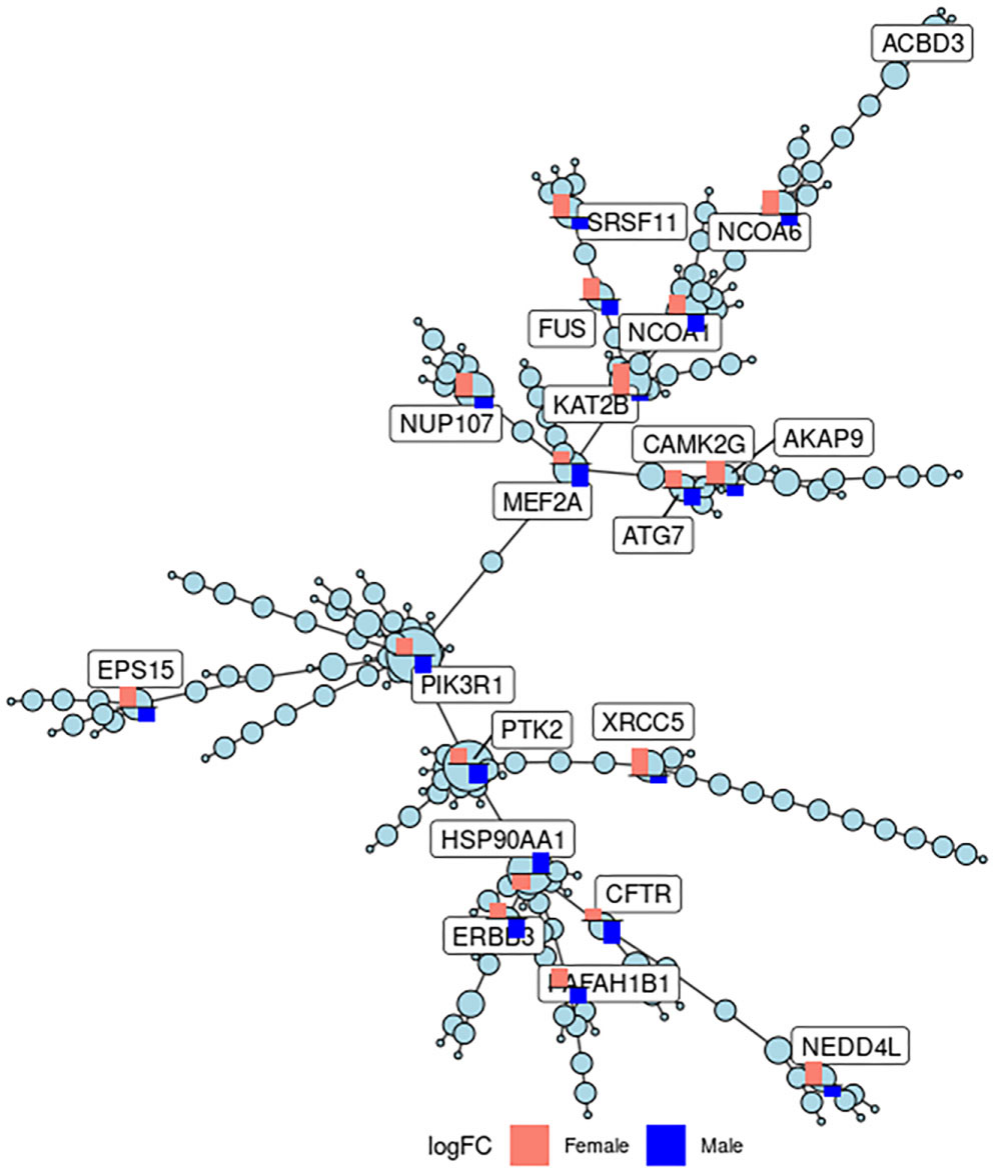
Hormone-influenced AKT-centered network in AD oligodendrocytes. The STRING-derived subnetwork highlights *PIK3R1* as a central hub connecting PI3K–AKT signaling with calcium-dependent pathways, cytoskeletal regulators, and proteostasis modules. For clarity, only the top 20 genes ranked by degree centrality are labeled.

To further contextualize these findings, we built a larger sub-network by mapping the same dimorphic genes onto the STRING PPI network (only interactions with a minimum combined score of 700 were included; see Fig. 3). Within this expanded network, *PIK3R1* exhibited the highest betweenness centrality (normalized score = 0.62), supporting its role as a key integrator of upstream signals. In addition to PI3K–AKT signaling, this sub-network highlighted dysregulation of interconnected pathways, including mTOR signaling, calcium-dependent signaling (e.g. *CAMK2G*), cytoskeletal and adhesion signaling (e.g. *PTK2*), and proteostasis and autophagy-related processes.

Taken together, these analyses indicate that sex-dimorphic genes in oligodendrocytes converge on a set of interconnected signaling pathways centered on PI3K–AKT. The consistent identification of *PIK3R1* as a central node across both sub-networks, combined with the observed directionality of expression changes, supports the interpretation of a sex-dependent shift in pathway activity rather than isolated gene-level effects. Within this framework, hormonal influences are likely to act upstream of these signaling cascades, contributing to differential regulation of pathways involved in metabolism, stress response, and cellular maintenance in AD.

### Performance benchmarking and scalability

Reproducibility and scalability are essential requirements for bioinformatics software in large-scale, biomedical omics analysis settings. We have conducted benchmarking of XYomics to ensure it performs efficiently across the spectrum of dataset sizes typical in current biomedical research.

#### Benchmark setup

Benchmarks were performed on a standard mid-range workstation to demonstrate accessibility (Intel Core i7-9700K CPU @ 3.60GHz, 8 Cores, 16 GB DDR4 RAM, running Ubuntu 20.04 LTS). We used synthetic single-cell datasets generated in R to test varying dimensions.

#### Runtime and memory analysis

**Single-cell scalability:** Runtime scales linearly with the number of cells. For 10 000 cells, the complete pipeline required approximately 110 s. For 50 000 cells, runtime was approximately 280 s ($< 5$ min). For 100 000 cells, runtime was approximately 600 s (10 min).

This linear scaling is achieved through the use of optimized matrix operations in R and the efficient implementation of the Wilcoxon test in the presto or Seurat backends.

**Memory footprint:** The package is memory-efficient. Peak RAM usage for the 50 000-cell dataset analysis (approximately 15 000 genes) was 8.2 GB. This confirms that XYomics can handle large-scale atlas-level data on standard laboratory workstations without requiring dedicated servers.

**Bulk omics performance:** For bulk datasets (e.g. RNA-seq counts and proteomics intensity tables) with $N=20$ to $N=200$ samples, the entire analysis workflow, including the computationally intensive PCSF network optimization, completes in under 90 s.

### Comparison with related software

To position XYomics within the existing software ecosystem, we performed a qualitative feature comparison with commonly used R packages for differential expression and enrichment analysis (Table 1).

**Table 1.** Comparison of integrated workflow features in XYomics and commonly used bioinformatics packages. “Int.” denotes integration

Package	Sex	Pathway	Hormone	Report
	Int.	Int.	Net.	Auto
limma	Partial	No	No	No
DESeq2	Partial	No	No	No
Seurat	Partial	No	No	No
clusterProfiler	No	Yes	No	No
RaNA-Seq	No	Yes	No	No
XYomics	Yes	Yes	Yes	Yes

While packages such as limma and DESeq2 provide the statistical approaches (e.g. linear models) that underpin transcriptomics, they function as low-level toolkits. They do not enforce specific workflows for SABV compliance, nor do they provide biological context. Conversely, pathway analysis tools such as clusterProfiler are suitable for general enrichment analysis but lack the logic to handle sex-dependent analyses (where sex-specific and sex-dimorphic patterns should be analyzed separately from sex-neutral patterns). XYomics fills a gap by integrating these layers into a single, comprehensive object-oriented workflow that enforces best practices for sex-difference research.

## Discussion

For many complex diseases, sex differences can strongly influence disease risk, clinical manifestations, and underlying molecular mechanisms, creating a need for bioinformatics tools that go beyond simple adjustment for sex as a confounding variable. XYomics explicitly addresses this need by providing an end-to-end framework for the discovery and interpretation of sex-dependent molecular mechanisms in functional omics data. By automating dual-threshold categorization, interaction modeling, and sex-aware pathway analysis, XYomics offers standardized, statistically rigorous workflows that reduce common analytical errors documented in the literature, promoting rigor and reproducibility in sex-based research [3–5].

Our example analysis for AD demonstrates that standard statistical models inadvertently obscure sex-dimorphic biology. By averaging opposing effects, conventional GLMs failed to detect over 75% of the sex-dimorphic genes identified by XYomics in our AD case study. This has profound implications for reproducibility in biomedical research: if a mechanism is upregulated in males and downregulated in females, a mixed-sex validation cohort will likely fail to replicate findings from a single-sex pilot study, and vice versa. This can lead to misinterpretations and wasted resources.

The identification of a *PIK3R1*-centered signaling architecture in AD oligodendrocytes illustrates the ability of XYomics to extract coherent biological signals from sex-dimorphic gene sets. Rather than implicating a specific hormone receptor, the analysis consistently highlights the PI3K–AKT signaling pathway as a central axis, with *PIK3R1* emerging as a hub across both hormone-informed and STRING-derived subnetworks. The coordinated expression patterns of *PIK3R1, AKT3*, and associated chaperones (*HSPA1A, HSP90AA1*) are consistent with a sex-dependent shift in pathway activity.

By integrating dimorphic genes within a hormone-related interaction framework and a broader PPI network, XYomics provides a pathway-level view of how upstream signals may converge on shared intracellular regulators. This approach complements standard enrichment analyses by identifying central nodes and suggesting coordinated, sex-dependent modulation of signaling pathways in AD oligodendrocytes.

More broadly, while XYomics builds on established statistical approaches, its core contribution is a new analytical framework, integrating sex-disease interaction analysis, the dual-threshold categorization logic, and sex-aware pathway and network analyses informed by curated hormone signaling interactions, that cannot be replicated by simply running existing tools in sequence.

**Limitations:** Users should be aware that the “Stratified Analysis” mode relies on the absence of evidence in one sex to infer specificity. While our dual-threshold approach mitigates false positives, it cannot strictly prove that a gene is unchanged, only that there is no statistical evidence of change. Conversely, the “Interaction Analysis” mode is rigorous but requires sample sizes ($N > 20$–30 per group) that are often unavailable in pilot biomedical studies. For single-cell data, this requirement applies to the number of independent biological replicates (donors), not the number of cells: a dataset with many thousands of cells but few donors may pass a cell-level check while still lacking statistical power at the biological level, owing to pseudo-replication. To make this explicit, the input validation functions can verify donor-level sample sizes when a donor column is supplied (see Materials and methods), but the underlying limitation, that biological replication ultimately constrains power, cannot be removed by increasing cell numbers alone. In addition, the current version of XYomics is designed for the most common experimental design in sex-difference research: two-group comparisons stratified by sex. Extending the framework to multi-group designs would require different statistical models (e.g. ANOVA-based approach); however, intermediate results from XYomics can be exported for integration with external statistical pipelines.

**Future directions:** While XYomics already supports individual analysis of transcriptomic, proteomic, and metabolomic datasets, future development will focus on joint multi-omics integration (e.g. cross-validating sex-specific signatures across omics layers), longitudinal models to track how sex differences evolve over disease progression, and the extension of the framework to multi-group experimental designs.

## Supplementary Material

gkag759_Supplemental_File

## Data Availability

The single-cell RNA sequencing dataset analyzed in this article is available from the Gene Expression Omnibus (GEO) under accession number GSE138852. The preprocessed demonstration data are available in the GitLab repository and archived at Zenodo: https://zenodo.org/records/17623749.
