# Supplementary material for: XYomics: detecting sex-dependent molecular mechanisms in omics data
Source: Nucleic Acids Res. 2026 Jul 30;54(14):gkag759. doi: 10.1093/nar/gkag759 (PMC13421391; doi:10.1093/nar/gkag759)
Supplement: gkag759_Supplemental_File [file gkag759_supplemental_file.pdf]

Supplementary Materials

XYomics: Detecting Sex-Dependent Molecular Mechanisms in Omics Data

Sophie Le Bars[1], Mohamed Soudy[1], and Enrico Glaab[1,*]

[1]Biomedical Data Science Group, Luxembourg Centre for Systems Biomedicine (LCSB), University of Luxembourg, 6 Avenue du Swing, Belvaux, 4367, Luxembourg

*Corresponding author: enrico.glaab@uni.lu

Contents

Supplementary Tables

- Supplementary Table S1: Top differentially expressed genes in astrocytes 3
- Supplementary Table S2: Top differentially expressed genes in microglial cells 4
- Supplementary Table S3: Top differentially expressed genes in neurons 5
- Supplementary Table S4: Top differentially expressed genes in oligodendrocytes 6
- Supplementary Table S5: Top differentially expressed genes in oligodendrocyte precursor cells 7
- Supplementary Table S6: Pathway enrichment results for astrocytes 8
- Supplementary Table S7: Pathway enrichment results for microglial cells 9
- Supplementary Table S8: Pathway enrichment results for neurons 10
- Supplementary Table S9: Pathway enrichment results for oligodendrocytes 11
- Supplementary Table S10: Pathway enrichment results for oligodendrocyte precursor cells 12
- Supplementary Table S11: Summary of analysis results across cell types 12

Supplementary Figures

- Supplementary Figure S1: Volcano plots (Astrocyte, Microglia) 13
- Supplementary Figure S2: Volcano plots (Neuron, Oligodendrocyte) 14
- Supplementary Figure S3: Volcano plot (Oligodendrocyte precursor cell) 15
- Supplementary Figure S4: Pathway enrichment dot plots (Astrocyte, Microglia) 16
- Supplementary Figure S5: Pathway enrichment dot plots (Neuron, Oligodendrocyte) . 17
- Supplementary Figure S6: Pathway enrichment dot plot (Oligodendrocyte precursor cell) 18
- Supplementary Figure S7: PPI networks (Astrocyte, Microglia) 19
- Supplementary Figure S8: PPI networks (Neuron, Oligodendrocyte) 20
- Supplementary Figure S9: PPI network (Oligodendrocyte precursor cell) 21
- Supplementary Figure S10: Hormonal PPI networks (Astrocyte, Microglia) 22
- Supplementary Figure S11: Hormonal PPI networks (Neuron, Oligodendrocyte) 23

Supplementary Tables

Differentially Expressed Genes by Cell Type

Supplementary Table 1: Top 25 differentially expressed genes in astrocytes classified by XYomics. The table shows the sex-dependence category (DEG_Type), gene symbol, log fold-change and FDR-adjusted p-value for males and females. A total of 1,294 DEGs were identified in astrocytes.

DEG Type	Gene	Male logFC	Male FDR	Female logFC	Female FDR
male-specific	DNAJB1	2.458	6.88e-52	-0.355	6.25e-01
male-specific	AC074363.1	-1.551	8.82e-40	-0.320	5.95e-01
male-specific	CLDND1	2.375	2.33e-37	-0.693	6.33e-01
male-specific	FKBP4	3.150	1.21e-34	-0.470	6.51e-01
male-specific	SLC26A3	2.851	8.00e-32	0.378	6.12e-01
male-specific	PHLPP1	-1.119	2.92e-28	-0.324	6.37e-01
male-specific	SRGAP3	-2.156	2.58e-27	-0.283	9.37e-01
male-specific	SPDYE2	-4.262	1.76e-24	-0.294	9.64e-01
male-specific	FAM19A1	-2.666	2.18e-24	-0.363	7.09e-01
male-specific	SUMF1	-1.929	7.04e-21	-0.288	5.99e-01
male-specific	CTD-2537O9.1	-2.902	1.27e-20	-0.264	5.87e-01
male-specific	NKX6-2	2.611	4.04e-19	-0.663	6.30e-01
male-specific	CHI3L1	1.865	1.21e-18	-0.421	7.64e-01
male-specific	DCHS2	-2.891	6.56e-18	-0.318	6.05e-01
male-specific	FGGY	-2.341	1.10e-17	0.255	7.41e-01
male-specific	CH507-513H4.1	1.542	1.16e-17	-0.307	7.62e-01
male-specific	WDR17	-1.491	3.71e-16	-0.292	6.81e-01
male-specific	HDAC8	-1.479	1.83e-15	-0.300	8.44e-01
male-specific	PHACTR1	-1.076	3.80e-15	-0.409	5.91e-01
male-specific	AATK	1.708	2.23e-14	-0.470	9.79e-01
male-specific	SOX10	2.389	4.97e-14	-0.470	7.10e-01
male-specific	DENND1B	-1.938	4.97e-14	-0.255	6.77e-01
male-specific	TUBA1A	1.310	8.87e-14	-0.308	7.22e-01
male-specific	SYNGR2	2.287	1.07e-13	-0.318	6.97e-01
male-specific	MLC1	-1.234	2.41e-13	0.267	6.12e-01

Supplementary Table 2: Top 25 differentially expressed genes in microglial cells classified by XYomics. A total of 1,034 DEGs were identified in microglial cells.

DEG Type	Gene	Male logFC	Male FDR	Female logFC	Female FDR
male-specific	ZBTB20	-1.191	2.50e-14	-0.320	7.07e-01
male-specific	ITM2B	-1.143	1.72e-08	-0.310	7.96e-01
male-specific	RSRP1	-1.406	8.42e-08	-0.435	9.38e-01
male-specific	HSP90AB1	1.787	1.34e-07	-0.539	8.78e-01
male-specific	PDIA4	3.822	2.79e-07	0.631	9.17e-01
male-specific	AIG1	-1.349	5.79e-05	-0.299	7.11e-01
male-specific	ZNF692	2.537	2.95e-04	0.631	9.17e-01
male-specific	BCOR	2.004	3.05e-04	-0.369	9.08e-01
male-specific	ATP5EP2	2.652	3.24e-04	0.408	8.95e-01
male-specific	ZNF331	-3.200	4.26e-04	-0.592	6.66e-01
male-specific	ECHDC2	-2.579	4.48e-04	-0.329	8.90e-01
male-specific	STIP1	2.325	4.86e-04	-0.287	9.52e-01
male-specific	FOXO1	-1.678	6.18e-04	-0.369	8.37e-01
male-specific	SAMD12	-0.969	6.52e-04	-0.351	7.16e-01
male-specific	LPAR1	-1.082	6.71e-04	-0.754	6.92e-01
male-specific	HMGN3	-0.930	6.96e-04	-0.257	6.47e-01
male-specific	PRMT9	-4.218	8.32e-04	0.408	8.92e-01
male-specific	FAM118A	-2.164	8.62e-04	0.993	7.11e-01
male-specific	RABL6	2.411	1.05e-03	-0.369	7.23e-01
male-specific	PCM1	-0.590	1.33e-03	-0.485	9.38e-01
male-specific	SAT2	-1.814	1.48e-03	-0.369	8.08e-01
male-specific	NRIP1	-1.489	1.53e-03	0.479	9.86e-01
male-specific	CCDC88A	-0.642	1.66e-03	-0.537	9.61e-01
male-specific	RP11-296O14.3	-3.164	1.76e-03	0.631	8.16e-01
male-specific	RPL36	1.317	1.78e-03	-0.604	7.11e-01

Supplementary Table 3: Top 25 differentially expressed genes in neurons classified by XYomics. A total of 692 DEGs were identified in neurons.

DEG Type	Gene	Male logFC	Male FDR	Female logFC	Female FDR
male-specific	QDPR	3.412	6.94e-58	-0.300	6.72e-01
male-specific	HSPB1	4.369	1.33e-41	-0.830	7.32e-01
male-specific	NPAS3	-1.617	1.17e-32	-0.364	7.04e-01
male-specific	CNP	3.030	5.39e-31	-0.468	6.77e-01
male-specific	THSD7A	-2.612	1.55e-28	-0.582	8.98e-01
male-specific	C8orf34	-2.509	2.17e-26	0.411	6.71e-01
male-specific	BIN1	2.337	2.07e-24	-0.333	6.92e-01
male-specific	PDE10A	-1.906	7.49e-21	0.298	6.57e-01
male-specific	GAD1	-2.572	7.95e-21	-0.349	6.57e-01
male-specific	OLIG1	3.687	4.68e-20	-0.830	9.36e-01
male-specific	UNC5D	-1.650	2.48e-19	-0.300	6.57e-01
male-specific	KIAA0930	2.169	1.23e-18	-0.567	6.59e-01
male-specific	FKBP4	2.371	2.44e-15	-0.830	7.32e-01
male-specific	PIP4K2A	2.326	5.97e-14	-0.415	6.61e-01
male-specific	CD81	2.219	7.32e-14	-0.830	7.40e-01
male-specific	RP11-405A12.2	-3.089	1.49e-13	-0.283	6.57e-01
male-specific	CACNA2D3	-1.412	2.62e-13	0.302	7.73e-01
male-specific	NXPH1	-1.670	2.77e-13	-0.330	8.59e-01
male-specific	SEPT8	2.398	3.20e-13	-0.678	7.62e-01
male-specific	MAPK8IP1	2.197	3.61e-13	-0.608	6.41e-01
male-specific	ENTPD3	-2.926	5.30e-12	0.299	7.11e-01
male-specific	KLF12	-1.343	7.22e-12	-0.263	6.66e-01
male-specific	MAN2A1	1.827	1.62e-11	-0.316	9.44e-01
male-specific	TPD52L2	2.079	1.98e-11	0.307	6.57e-01
male-specific	UBC	1.499	2.55e-11	-0.268	6.57e-01

Supplementary Table 4: Top 25 differentially expressed genes in oligodendrocytes classified by XYomics. A total of 2,614 DEGs were identified in oligodendrocytes, the highest count among all cell types analyzed.

DEG Type	Gene	Male logFC	Male FDR	Female logFC	Female FDR
male-specific	GFAP	3.877	2.52e-233	-0.287	9.24e-01
male-specific	DNAJB2	2.043	1.01e-122	-0.488	6.08e-01
male-specific	MT-ATP6	2.418	4.95e-110	-0.362	7.85e-01
male-specific	MT-CYB	2.199	4.99e-102	-0.320	6.26e-01
male-specific	DSCAML1	-1.533	1.64e-87	-0.277	5.59e-01
male-specific	MAGI1	-1.329	1.21e-69	-0.294	9.75e-01
male-specific	PLXDC2	-0.798	2.74e-66	-0.293	9.11e-01
male-specific	RP11-50D16.4	-1.570	5.92e-61	-0.288	8.33e-01
male-specific	STIP1	2.785	2.09e-60	-0.277	9.65e-01
male-specific	PLPPR1	-1.204	3.63e-53	-0.325	7.24e-01
male-specific	SEPT4	-0.845	1.09e-52	-0.439	6.06e-01
male-specific	MYO5A	-1.101	1.58e-51	-0.284	8.71e-01
male-specific	PBX1	-1.147	4.36e-44	-0.304	6.35e-01
male-specific	IMMP2L	-1.041	5.69e-40	-0.309	8.41e-01
male-specific	GRAMD3	-1.265	6.85e-40	-0.330	7.07e-01
male-specific	NSF	-1.445	1.14e-28	-0.431	8.50e-01
male-specific	SLC12A2	-0.604	1.01e-27	-0.352	6.46e-01
male-specific	FOS	2.371	2.37e-27	-0.459	6.13e-01
male-specific	KIDINS220	-0.702	1.54e-26	-0.298	9.80e-01
male-specific	COLGALT2	-0.554	1.89e-25	-0.474	6.34e-01
male-specific	SCGB2B2	-1.819	2.49e-24	0.263	7.36e-01
male-specific	INF2	1.341	2.84e-22	-0.411	5.39e-01
male-specific	RPS13	-1.097	7.22e-21	-0.289	8.04e-01
male-specific	RHOU	-0.808	1.97e-20	-0.341	7.01e-01
male-specific	C10orf11	-1.700	3.08e-20	-0.723	6.02e-01

Supplementary Table 5: Top 25 differentially expressed genes in oligodendrocyte precursor cells (OPCs) classified by XYomics. A total of 619 DEGs were identified in OPCs.

DEG Type	Gene	Male logFC	Male FDR	Female logFC	Female FDR
male-specific	DNAJB2	3.689	9.43e-70	-0.304	6.84e-01
male-specific	HSPB1	2.981	2.60e-39	-0.430	8.59e-01
male-specific	CNTN2	2.777	1.80e-37	0.418	6.71e-01
male-specific	DHCR24	3.820	5.36e-24	0.570	8.01e-01
male-specific	NTRK2	-1.240	1.40e-21	-0.290	6.63e-01
male-specific	SGCD	-0.966	9.45e-19	-0.278	7.19e-01
male-specific	RP11-436D23.1	-0.986	1.03e-17	-0.313	6.79e-01
male-specific	PDE4D	-0.714	2.01e-17	-0.267	7.19e-01
male-specific	MAGI1	-1.252	3.03e-13	-0.319	6.65e-01
male-specific	INF2	2.035	4.56e-13	0.570	7.19e-01
male-specific	AC010127.3	-2.739	7.28e-13	0.307	9.07e-01
male-specific	HP1BP3	1.409	1.75e-12	-0.260	6.57e-01
male-specific	KIAA1211	-1.866	3.85e-12	0.492	6.57e-01
male-specific	AHNAK	3.556	5.09e-12	-0.430	6.57e-01
male-specific	CA8	-2.143	7.37e-12	-0.508	7.22e-01
male-specific	DTNA	-0.863	9.58e-12	-0.405	6.57e-01
male-specific	ASTN1	-1.207	3.85e-11	-0.292	7.19e-01
male-specific	CACNB2	-1.734	6.11e-11	-0.430	7.32e-01
male-specific	PLOD3	1.999	1.31e-10	0.307	8.04e-01
male-specific	MOBP	1.722	1.62e-10	-0.567	7.10e-01
male-specific	RUNX1T1	-1.111	2.79e-10	-0.387	8.01e-01
male-specific	KCTD8	-1.309	4.96e-10	-0.266	9.92e-01
male-specific	JADE2	2.539	9.63e-10	0.570	8.01e-01
male-specific	MAP2	-0.524	2.46e-09	-0.260	7.17e-01
male-specific	PHLPP1	-0.648	2.81e-09	-0.382	8.01e-01

Pathway Enrichment Results by Cell Type

Supplementary Table 6: Top pathway enrichment results for astrocytes. Pathways are ranked by adjusted p-value. A total of 30 significantly enriched pathways were identified.

DEG Type	Category	ID	Description	Gene Ratio	p.adjust	Count
female_specific	Transport/catabolism	hsa04144	Endocytosis	3/7	1.76e-02	3
sex_dimorphic	Endocrine system	hsa04911	Insulin secretion	10/210	5.43e-03	10
sex_dimorphic	Nervous system	hsa04720	Long-term potentiation	8/210	1.03e-02	8
sex_dimorphic	Endocrine system	hsa04922	Glucagon signaling pathway	10/210	1.03e-02	10
sex_dimorphic	Endocrine system	hsa04921	Oxytocin signaling pathway	12/210	1.03e-02	12
sex_dimorphic	Endocrine system	hsa04912	GnRH signaling pathway	9/210	1.03e-02	9
sex_dimorphic	Endocrine system	hsa04918	Thyroid hormone synthesis	8/210	1.03e-02	8
sex_dimorphic	Digestive system	hsa04971	Gastric acid secretion	8/210	1.03e-02	8
sex_dimorphic	Digestive system	hsa04970	Salivary secretion	9/210	1.03e-02	9
sex_dimorphic	Digestive system	hsa04978	Mineral absorption	7/210	1.19e-02	7
sex_dimorphic	Transport/catabolism	hsa04144	Endocytosis	15/210	1.38e-02	15
sex_dimorphic	Circulatory system	hsa04270	Vascular smooth muscle contraction	10/210	2.01e-02	10
sex_dimorphic	Cellular community	hsa04520	Adherens junction	8/210	2.46e-02	8
sex_dimorphic	Excretory system	hsa04960	Aldosterone-regulated Na reabsorption	5/210	2.96e-02	5
sex_dimorphic	Endocrine system	hsa04915	Estrogen signaling pathway	9/210	4.53e-02	9

Supplementary Table 7: Top pathway enrichment results for microglial cells. Pathways are ranked by adjusted p-value. A total of 35 significantly enriched pathways were identified, with strong enrichment for immune-related pathways.

DEG Type	Category	ID	Description	Gene Ratio	p.adjust	Count
sex_dimorphic	Immune disease	hsa05330	Allograft rejection	9/269	2.94e-04	9
sex_dimorphic	Metabolic disease	hsa04940	Type I diabetes mellitus	9/269	3.53e-04	9
sex_dimorphic	Immune disease	hsa05332	Graft-versus-host disease	9/269	3.53e-04	9
sex_dimorphic	Infectious disease	hsa05168	Herpes simplex virus 1 infection	17/269	8.63e-04	17
sex_dimorphic	Immune disease	hsa05320	Autoimmune thyroid disease	9/269	8.63e-04	9
sex_dimorphic	Immune system	hsa04612	Antigen processing and presentation	11/269	8.63e-04	11
sex_dimorphic	Cardiovascular	hsa05416	Viral myocarditis	10/269	1.02e-03	10
sex_dimorphic	Immune system	hsa04664	Fc epsilon RI signaling pathway	9/269	4.67e-03	9
sex_dimorphic	Signaling molecules	hsa04514	Cell adhesion molecules	14/269	5.54e-03	14
sex_dimorphic	Immune system	hsa04611	Platelet activation	12/269	5.54e-03	12
sex_dimorphic	Immune disease	hsa05310	Asthma	6/269	5.54e-03	6
sex_dimorphic	Immune system	hsa04662	B cell receptor signaling pathway	10/269	5.54e-03	10
sex_dimorphic	Immune system	hsa04659	Th17 cell differentiation	11/269	5.54e-03	11
sex_dimorphic	Immune system	hsa04658	Th1 and Th2 cell differentiation	10/269	5.82e-03	10
sex_dimorphic	Transport/catabolism	hsa04140	Autophagy - animal	14/269	5.91e-03	14

Supplementary Table 8: Pathway enrichment results for neurons. A total of 25 significantly enriched pathways were identified, including both sex-dimorphic and sex-neutral categories.

DEG Type	Category	ID	Description	Gene Ratio	p.adjust	Count
sex_dimorphic	Endocrine system	hsa04915	Estrogen signaling pathway	4/18	1.17e-02	4
sex_dimorphic	Cell growth/death	hsa04217	Necroptosis	4/18	1.17e-02	4
sex_dimorphic	Nervous system	hsa04720	Long-term potentiation	3/18	1.17e-02	3
sex_dimorphic	Immune system	hsa04612	Antigen processing and presentation	3/18	1.46e-02	3
sex_dimorphic	Cardiovascular	hsa05417	Lipid and atherosclerosis	4/18	1.46e-02	4
sex_dimorphic	Cancer	hsa05207	Chemical carcinogenesis	4/18	1.46e-02	4
sex_dimorphic	Infectious disease	hsa05132	Salmonella infection	4/18	1.90e-02	4
sex_dimorphic	Endocrine system	hsa04914	Progesterone-mediated oocyte maturation	3/18	1.90e-02	3
sex_dimorphic	Nervous system	hsa04726	Serotonergic synapse	3/18	1.90e-02	3
sex_neutral	Signaling molecules	hsa04080	Neuroactive ligand-receptor interaction	8/21	5.81e-05	8
sex_neutral	Endocrine system	hsa04911	Insulin secretion	4/21	1.48e-03	4
sex_neutral	Hormone signaling	hsa04081	Hormone signaling	5/21	2.43e-03	5
sex_neutral	Signal transduction	hsa04024	cAMP signaling pathway	5/21	2.43e-03	5
sex_neutral	Nervous system	hsa04723	Retrograde endocannabinoid signaling	4/21	5.02e-03	4
sex_neutral	Digestive system	hsa04971	Gastric acid secretion	3/21	8.69e-03	3

Supplementary Table 9: Top pathway enrichment results for oligodendrocytes. A total of 40 significantly enriched pathways were identified, reflecting the high number of DEGs in this cell type.

DEG Type	Category	ID	Description	Gene Ratio	p.adjust	Count
sex_dimorphic	Transport/catabolism	hsa04144	Endocytosis	45/777	1.55e-04	45
sex_dimorphic	Folding/degradation	hsa04120	Ubiquitin mediated proteolysis	30/777	1.88e-04	30
sex_dimorphic	Transport/catabolism	hsa04140	Autophagy - animal	31/777	1.82e-03	31
sex_dimorphic	Signal transduction	hsa04150	mTOR signaling pathway	29/777	2.51e-03	29
sex_dimorphic	Drug resistance	hsa01521	EGFR TKI resistance	18/777	3.89e-03	18
sex_dimorphic	Cell growth/death	hsa04218	Cellular senescence	28/777	3.89e-03	28
sex_dimorphic	Aging	hsa04213	Longevity regulating pathway	15/777	5.23e-03	15
sex_dimorphic	Folding/degradation	hsa04141	Protein processing in ER	29/777	5.89e-03	29
sex_dimorphic	Translation	hsa03013	Nucleocytoplasmic transport	21/777	5.89e-03	21
sex_dimorphic	Nervous system	hsa04722	Neurotrophin signaling pathway	22/777	9.06e-03	22
sex_dimorphic	Endocrine system	hsa04910	Insulin signaling pathway	24/777	9.30e-03	24
sex_dimorphic	Signal transduction	hsa04070	Phosphatidylinositol signaling	19/777	9.30e-03	19
sex_dimorphic	Amino acid metabolism	hsa00280	Val, Leu, Ile degradation	12/777	9.77e-03	12
sex_dimorphic	Metabolic disease	hsa04931	Insulin resistance	20/777	1.19e-02	20
sex_dimorphic	Signal transduction	hsa04012	ErbB signaling pathway	17/777	1.19e-02	17

Supplementary Table 10: Pathway enrichment results for oligodendrocyte precursor cells. Only 2 significantly enriched pathways were identified, both in the sex-neutral category related to lipid metabolism.

DEG Type	Category	ID	Description	Gene Ratio	p.adjust	Count
sex_neutral	Digestive system	hsa04979	Cholesterol metabolism	2/12	4.27e-02	2
sex_neutral	Lipid metabolism	hsa00561	Glycerolipid metabolism	2/12	4.27e-02	2

Supplementary Table 11: Summary of XYomics analysis results across cell types in Alzheimer's disease single-cell RNA-seq data.

Cell Type	Total DEGs	Enriched Pathways
Astrocyte	1,294	30
Microglial cell	1,034	35
Neuron	692	25
Oligodendrocyte	2,614	40
Oligodendrocyte precursor cell	619	2
**Total**	**6,253**	**132**

Supplementary Figures

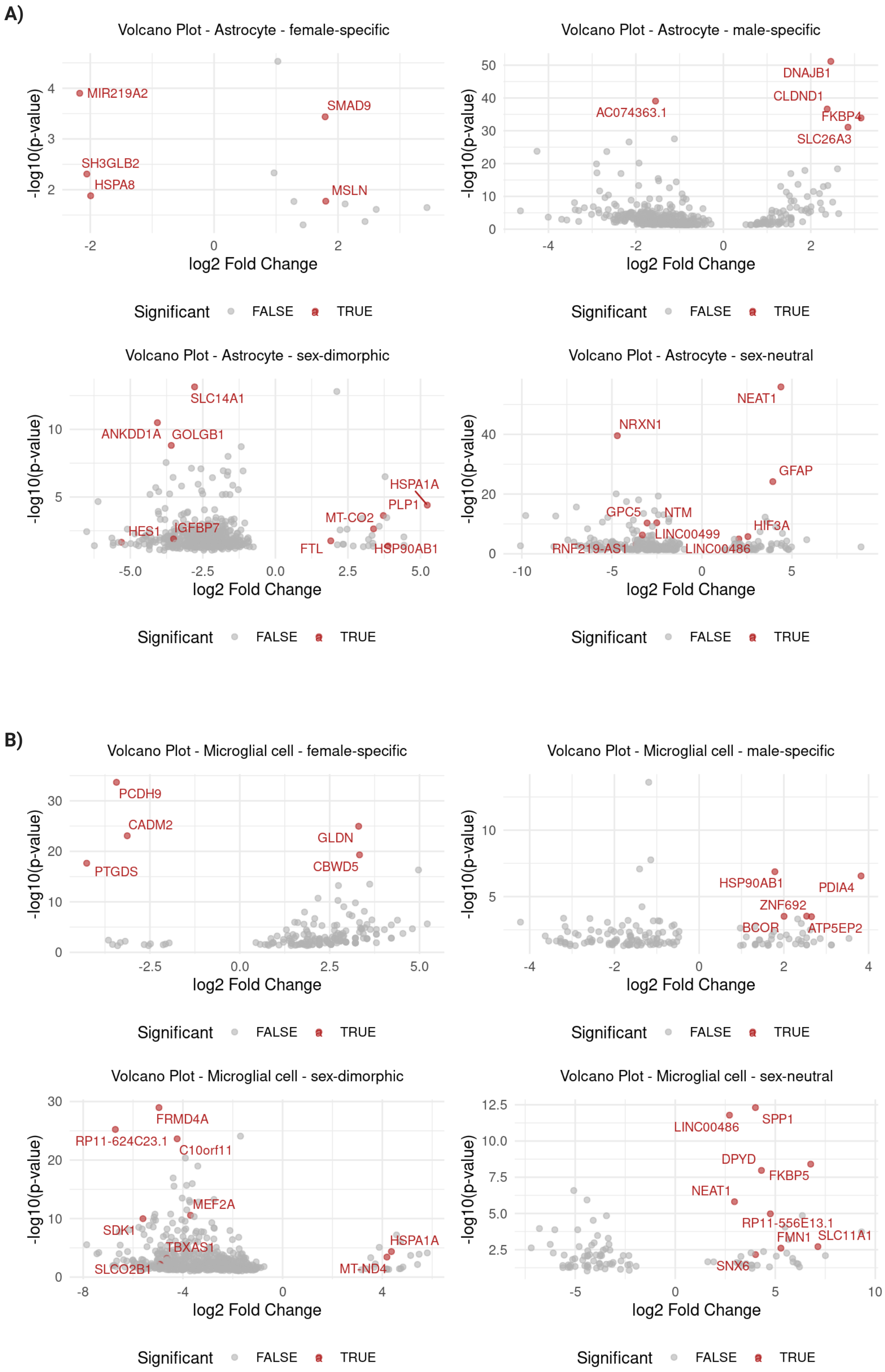

Supplementary Figure 1: Volcano plots of sex-categorized differentially expressed genes in astrocytes and microglial cells. Each panel shows the relationship between log fold-change (x-axis) and statistical significance (-log10 FDR, y-axis) for a specific sex-dependence category. Points are colored by significance status (red = significant, gray = non-significant). Separate panels are shown for female-specific, male-specific, sex-dimorphic, and sex-neutral genes. (A) Astrocytes (n=1,294 DEGs), (B) Microglial cells (n=1,034 DEGs).

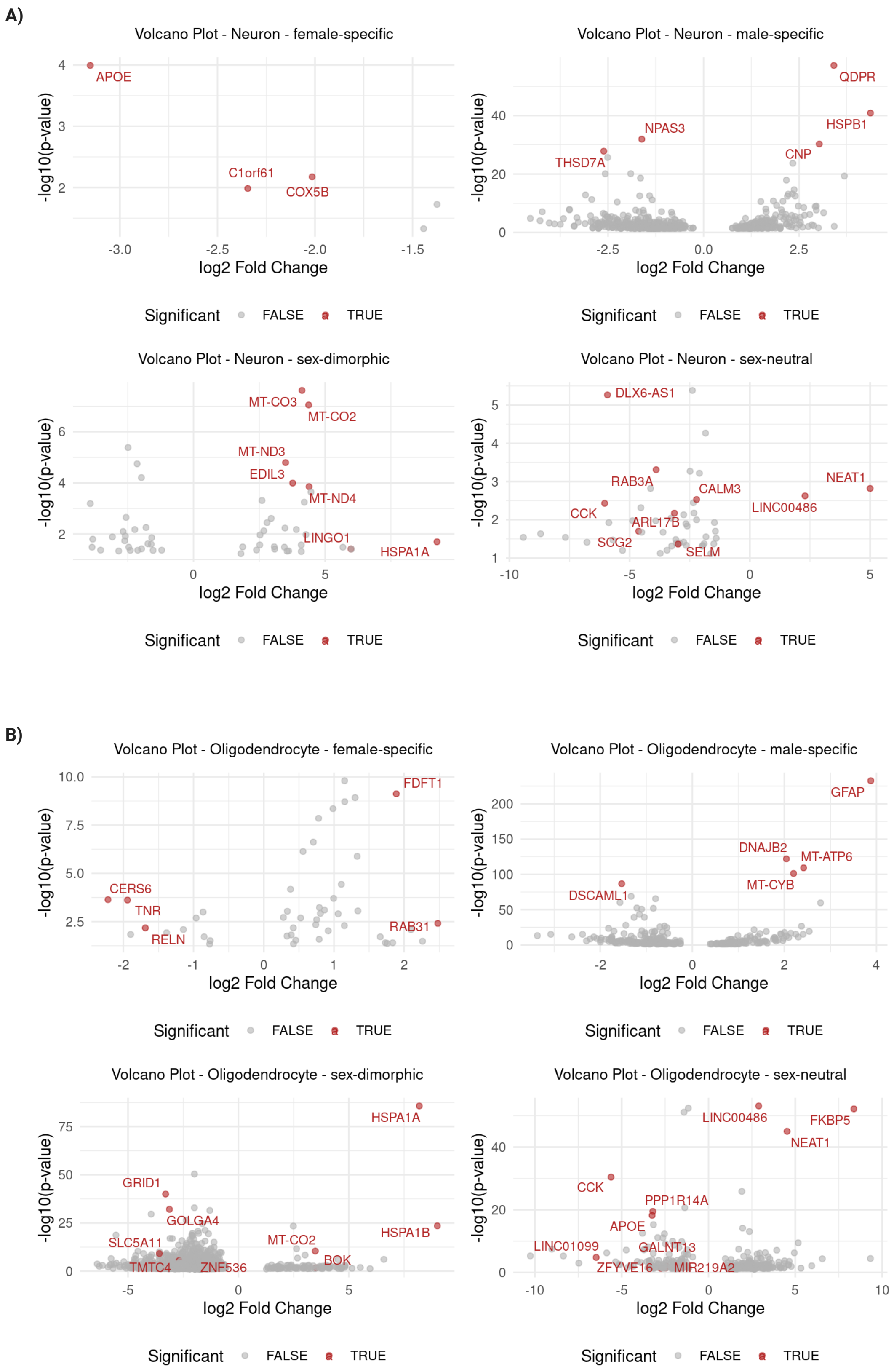

Supplementary Figure 2: Volcano plots of sex-categorized differentially expressed genes in neurons and oligodendrocytes. Points are colored by significance status (red = significant, gray = non-significant). Separate panels are shown for female-specific, male-specific, sex-dimorphic, and sex-neutral genes. (A) Neurons (n=692 DEGs), (B) Oligodendrocytes (n=2,614 DEGs).

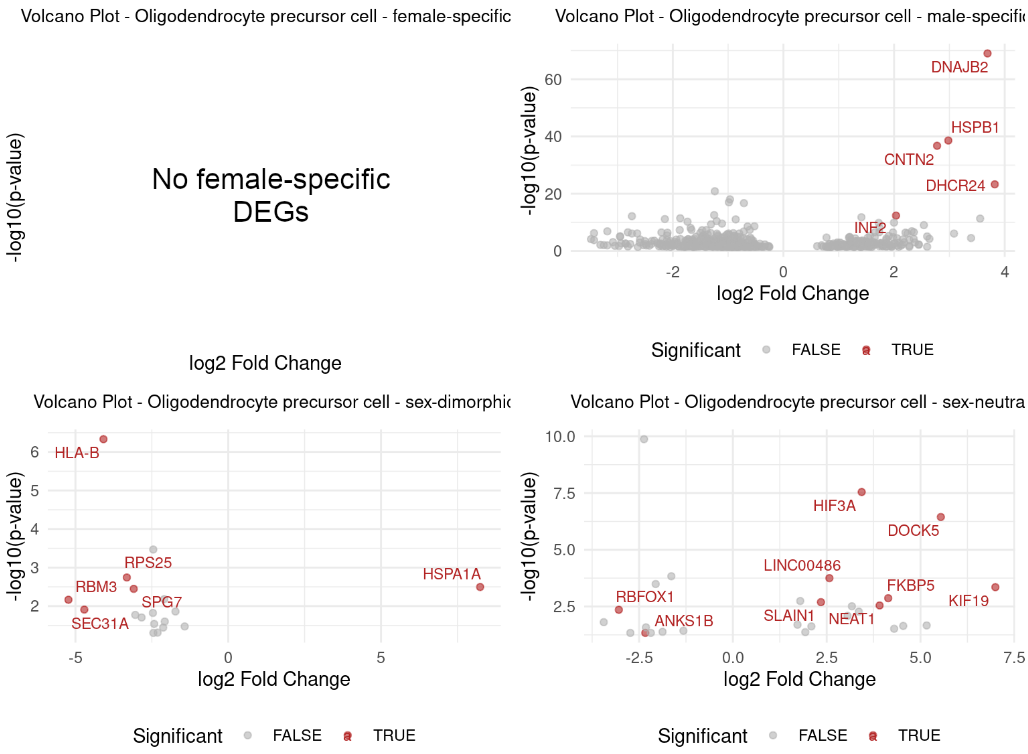

Supplementary Figure 3: Volcano plot of sex-categorized differentially expressed genes in oligodendrocyte precursor cells (OPCs). Points are colored by significance status (red = significant, gray = non-significant). Separate panels are shown for female-specific, male-specific, sex-dimorphic, and sex-neutral genes. A total of 619 DEGs were identified in OPCs.

A)

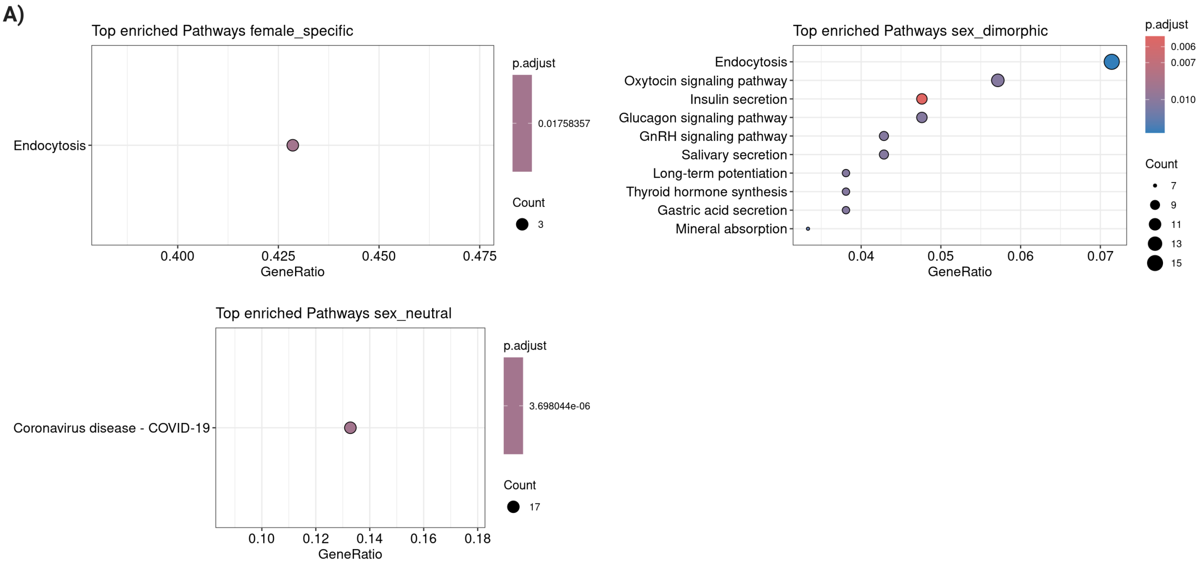

B)

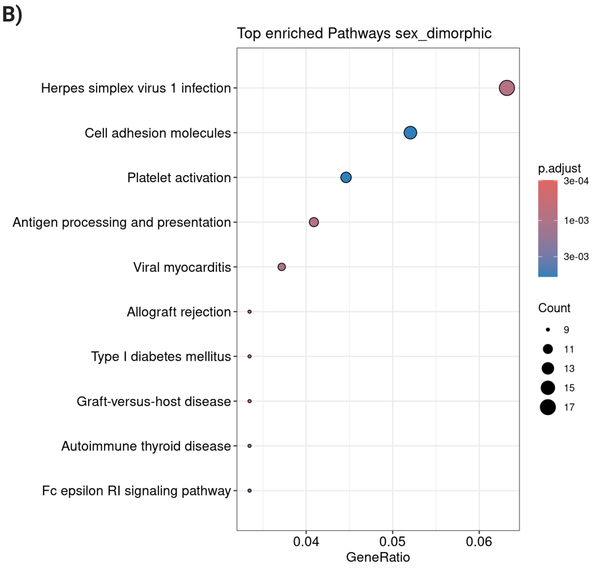

Supplementary Figure 4: Pathway enrichment dot plots for astrocytes and microglial cells. Each dot represents a significantly enriched KEGG pathway. Dot size indicates the gene count (number of DEGs in the pathway), and color indicates the adjusted p-value (darker colors represent more significant enrichment). Pathways are grouped by sex-dependence category of the constituent DEGs. (A) Astrocytes showing enrichment in endocrine and hormone signaling pathways, (B) Microglial cells showing strong enrichment in immune-related pathways.

A)

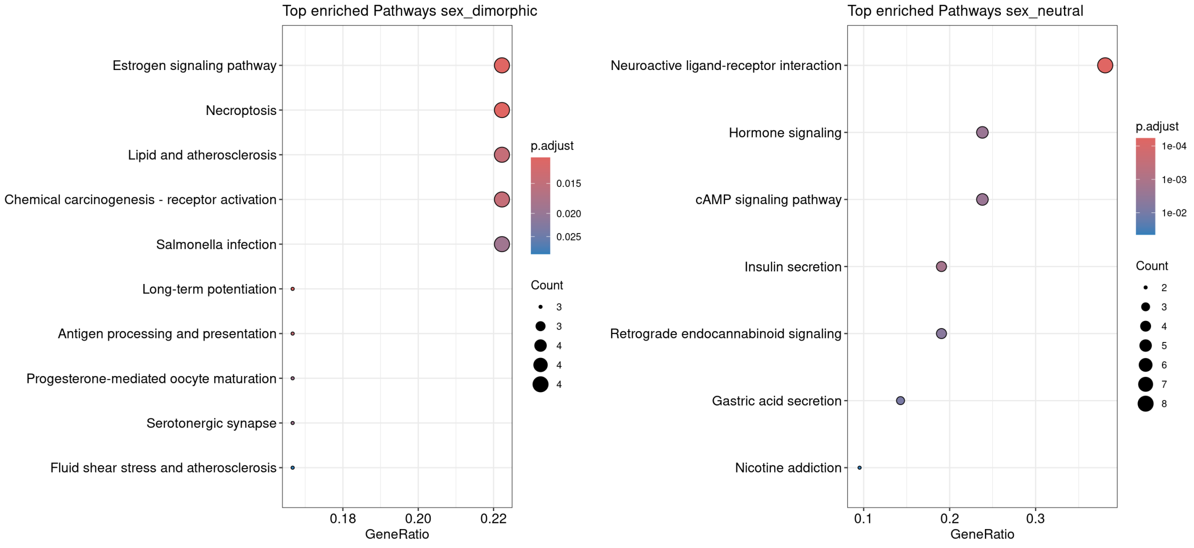

B)

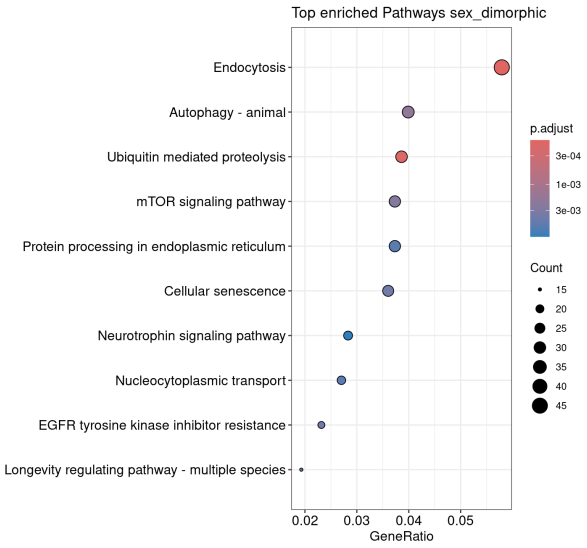

Supplementary Figure 5: Pathway enrichment dot plots for neurons and oligodendrocytes. Dot size indicates gene count, and color indicates adjusted p-value. (A) Neurons showing enrichment in neuronal signaling pathways, (B) Oligodendrocytes showing enrichment in cellular transport and metabolism pathways.

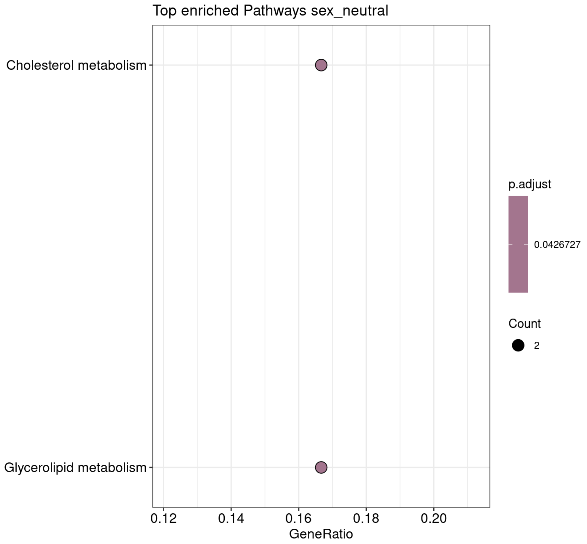

Supplementary Figure 6: Pathway enrichment dot plot for oligodendrocyte precursor cells (OPCs). Only 2 significantly enriched pathways were identified, both in the sex-neutral category related to lipid metabolism (cholesterol metabolism and glycerolipid metabolism).

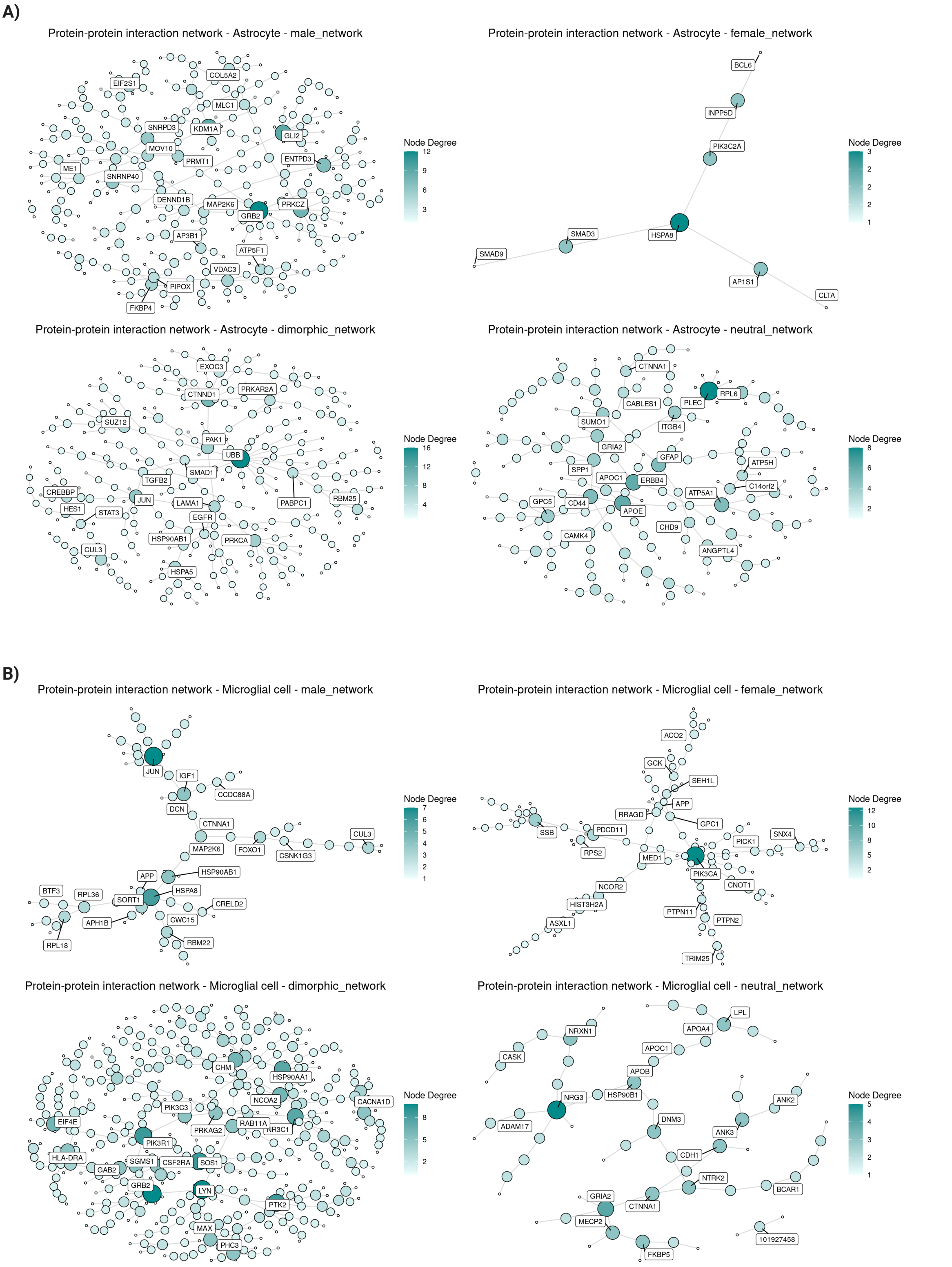

Supplementary Figure 7: Protein-protein interaction (PPI) networks for astrocytes and microglial cells. Networks were constructed from sex-categorized DEGs using the XYomics integrated network analysis module. Separate panels are shown for male-specific, female-specific, sex-dimorphic, and sex-neutral networks. Node color intensity reflects network centrality (degree), with darker nodes having more connections. Edges represent known or predicted protein-protein interactions from the STRING database. (A) Astrocyte PPI networks, (B) Microglial cell PPI networks.

A)

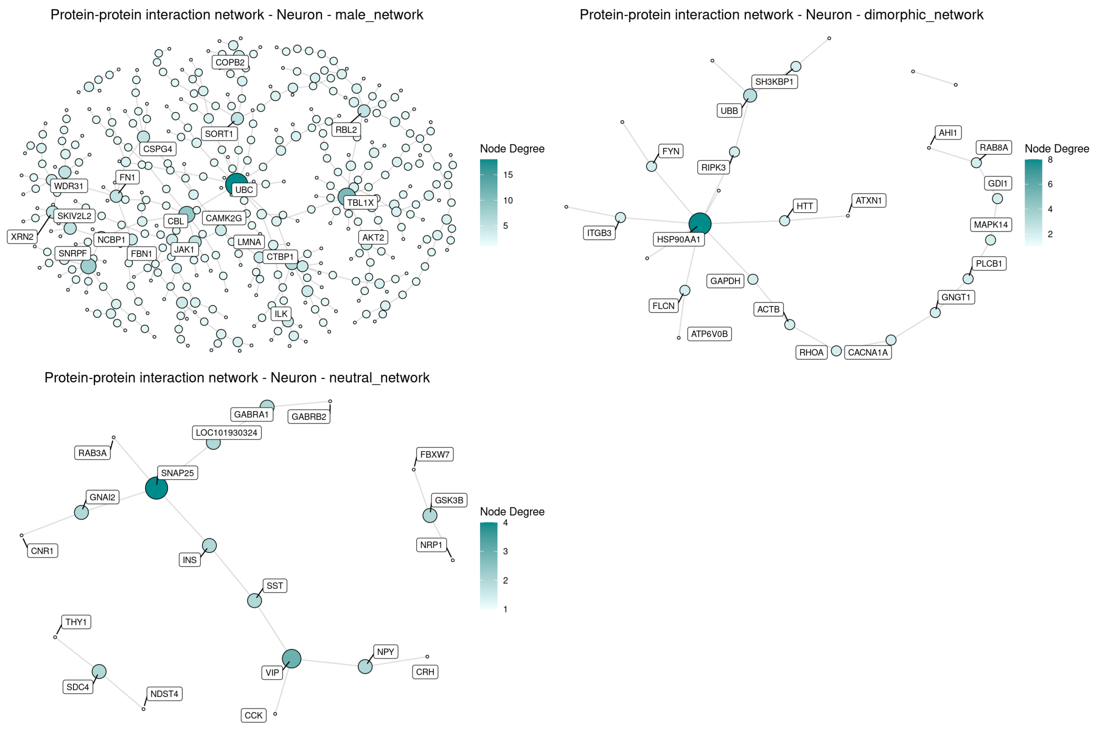

B)

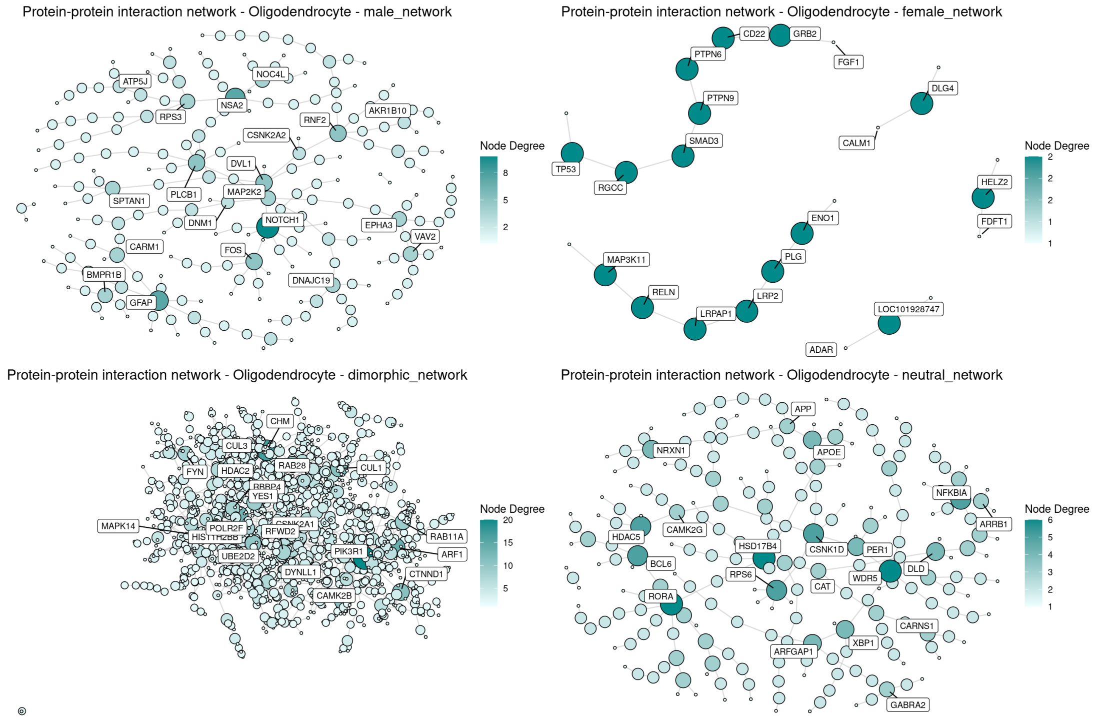

Supplementary Figure 8: Protein-protein interaction (PPI) networks for neurons and oligodendrocytes. Separate panels are shown for male-specific, female-specific, sex-dimorphic, and sex-neutral networks. Node color intensity reflects network centrality (degree). Edges represent protein-protein interactions from the STRING database. (A) Neuron PPI networks, (B) Oligodendrocyte PPI networks.

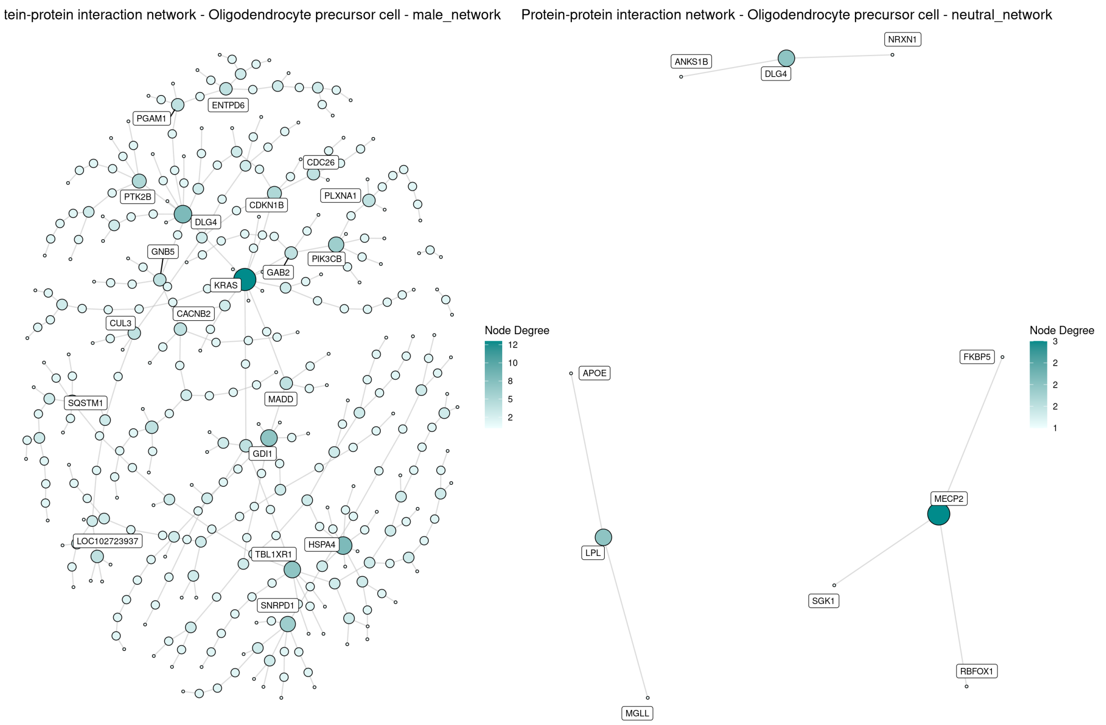

Supplementary Figure 9: Protein-protein interaction (PPI) network for oligodendrocyte precursor cells (OPCs). Separate panels are shown for male-specific and sex-neutral networks. Node color intensity reflects network centrality (degree). Edges represent protein-protein interactions from the STRING database.

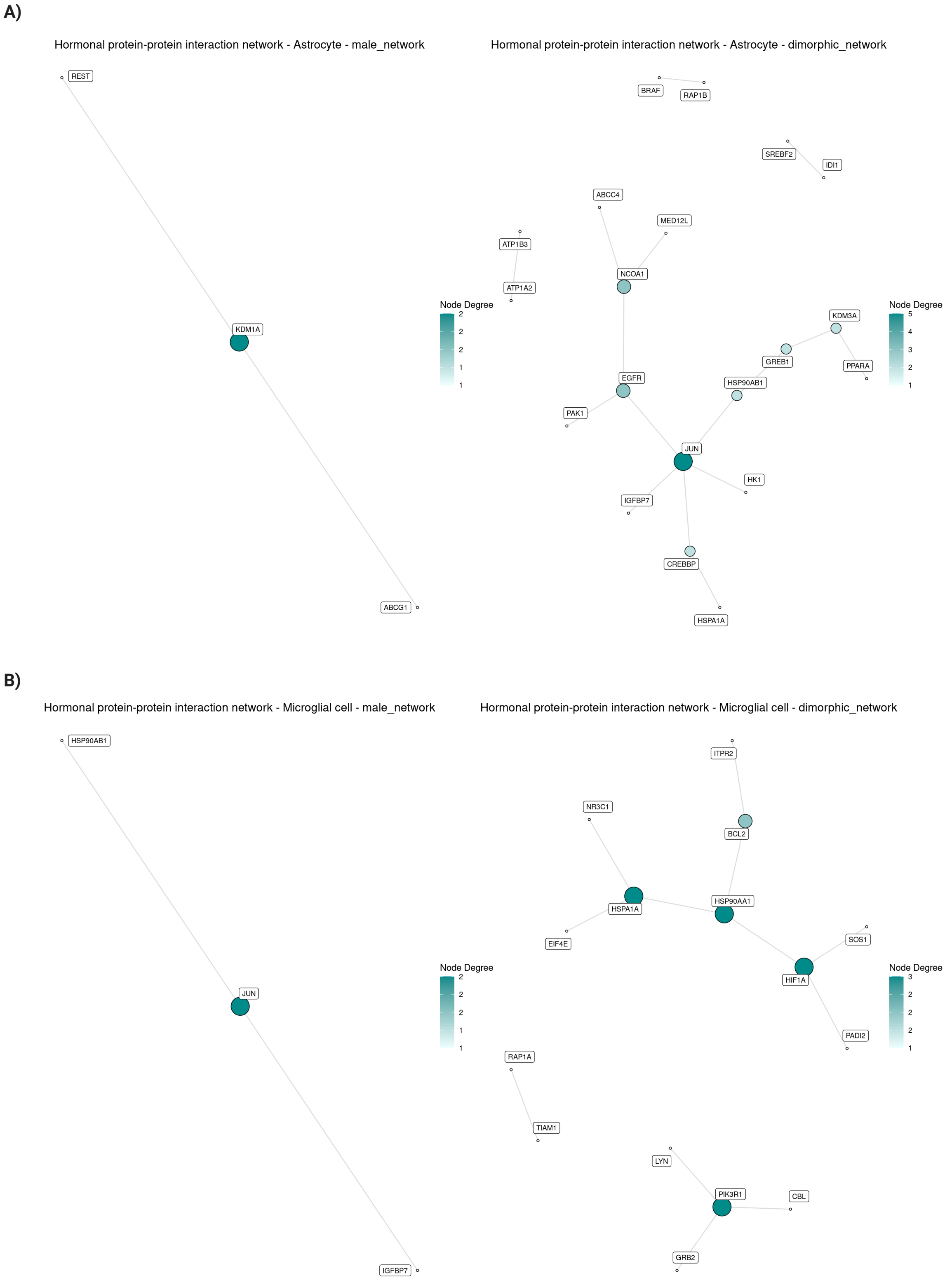

Supplementary Figure 10: Hormonal protein-protein interaction (PPI) networks for astrocytes and microglial cells. Networks were constructed by integrating sex-categorized DEGs with the XYomics curated hormone signaling interactome. Separate panels are shown for male-specific and sex-dimorphic networks. Node color intensity reflects network centrality (degree). Edges represent experimentally validated or literature-curated hormone-mediated interactions. (A) Astrocyte hormonal PPI networks, (B) Microglial cell hormonal PPI networks.

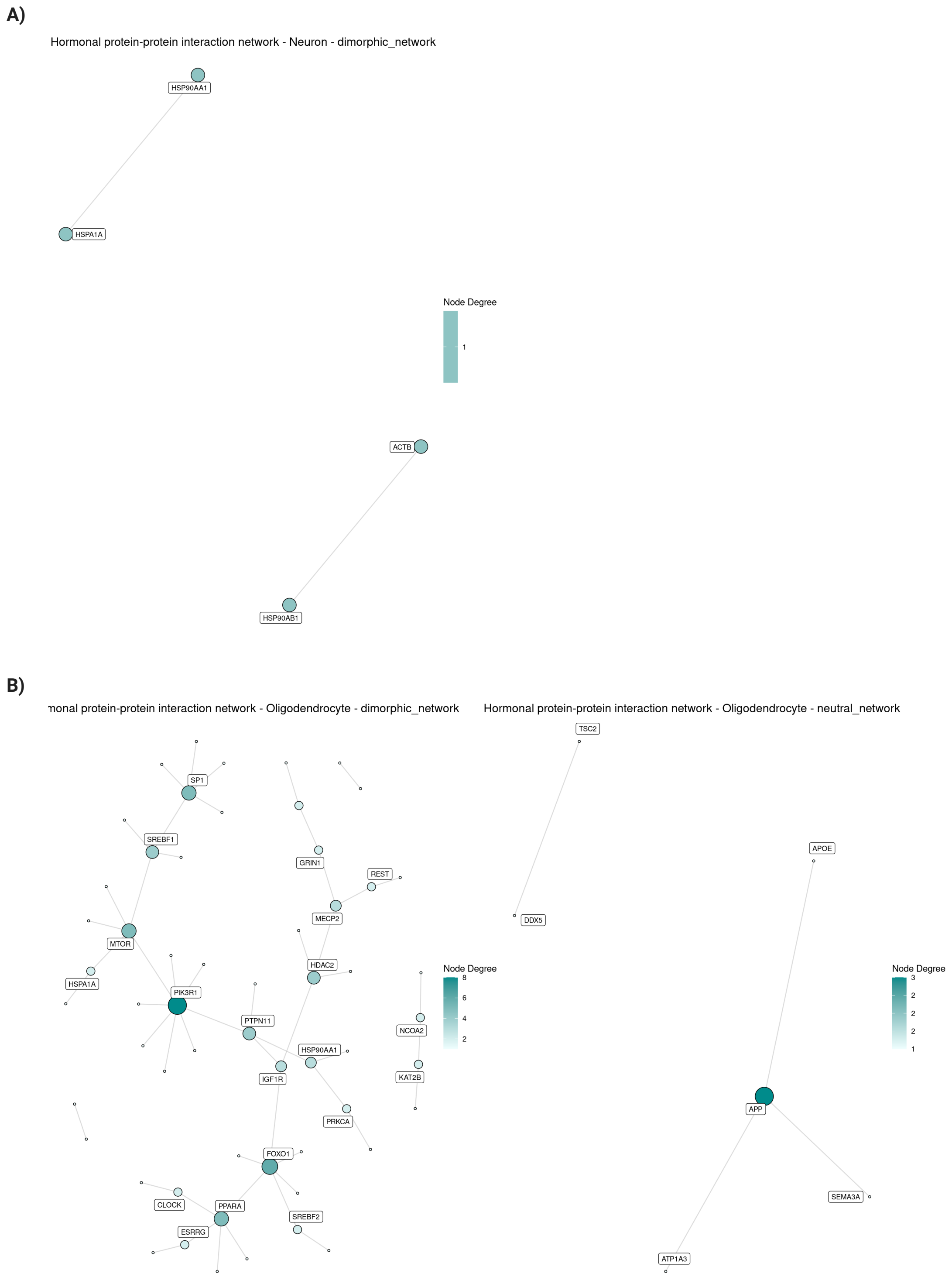

Supplementary Figure 11: Hormonal protein-protein interaction (PPI) networks for neurons and oligodendrocytes. Networks highlight sex-dependent DEGs connected through hormone signaling pathways from the curated interactome. Separate panels are shown for sex-dimorphic and sex-neutral networks. Node color intensity reflects network centrality (degree), and edges represent hormone-mediated regulatory interactions. (A) Neuron hormonal PPI networks, (B) Oligodendrocyte hormonal PPI networks.

Data Availability

Complete tables of all differentially expressed genes, pathway enrichment results, and network analysis outputs are available in the supplementary data files deposited at the XYomics GitLab repository: https://gitlab.com/uniluxembourg/lcsb/bds/xyomics.
